# Changes in functional performance, lower-limb muscle activation, balance, and gait symmetry after a 12-week elastic band resistance training program in older adults with sarcopenia: a single-arm pre–post study

**DOI:** 10.3389/fphys.2026.1970041

**Published:** 2026-10-29

**Authors:** Zhengbin Li, Fei Gao, Yang Liu, Na Liu

**Affiliations:** School of Human Movement Science, Jilin Sport University, Changchun, China

**Keywords:** balance, elastic band resistance training, gait symmetry, sarcopenia, sit-to-stand, surface electromyography

## Abstract

**Background:**

Sarcopenia is characterized by progressive declines in skeletal muscle mass and physical function, resulting in impaired mobility, balance deficits, and gait abnormalities in older adults. Although elastic band resistance training has been reported to improve muscle strength and functional performance, its associations with sit-to-stand performance, task-related lower-limb electromyographic activity, balance function, and gait symmetry in older adults with sarcopenia remain insufficiently understood. This study evaluated pre–post changes associated with a 12-week elastic band resistance training program across these outcomes.

**Methods:**

A total of 120 community-dwelling older adults with sarcopenia were recruited, of whom 102 participants aged 61.5–73.1 years completed the 12-week intervention and post-intervention assessments. The primary outcome reported was the change in Five Times Sit-to-Stand Test (FTSST) completion time from baseline to 12 weeks. Secondary functional outcomes were Timed Up and Go Test (TUGT) and sit-to-stand transfer time. Lower-limb surface electromyography [task-normalized root mean square (RMS) and integrated electromyography (IEMG)], Berg Balance Scale (BBS), and gait symmetry indices (step length, stance phase, swing phase, and contact time) were assessed before and after the intervention. Pre- and post-intervention differences were analyzed using paired statistical tests with Holm adjustment within specified families of secondary outcomes.

**Results:**

At the post-intervention assessment, lower-limb functional performance was better, as reflected by reduced FTSST, TUGT, and sit-to-stand transfer time. RMS values were lower in all examined muscles, whereas IEMG values were higher only in the bilateral rectus femoris and tibialis anterior muscles after Holm adjustment. BBS scores were also higher after the program, indicating better performance on the balance scale. All gait symmetry indices were lower at the post-intervention assessment, indicating reduced inter-limb asymmetry during walking.

**Conclusion:**

Among the 102 participants who completed both baseline and post-intervention assessments, functional performance, balance scores, and gait symmetry measures improved over the 12-week exercise program. The uncontrolled design prevents causal attribution to the program or its individual components. Attrition and limitations of between-session EMG comparisons further constrain interpretation. Controlled studies are needed to investigate training effects and the persistence of the observed changes.

## 1 Introduction

As the global population continues to age, age-related health conditions have gained increasing prominence (von Haehling et al., 2010; Jane Osareme et al., 2024). Sarcopenia, a syndrome marked by the progressive decline of muscle mass, strength, and physical function, has emerged as a critical factor influencing morbidity and mortality among older adults (Larsson et al., 2019). Epidemiological studies indicate that initial muscle deterioration begins around the age of 40, with more rapid declines occurring after age 50. Specifically, lower extremity muscle mass decreases by 1%-2% annually, while muscle strength diminishes at a faster rate of 1.5%-5% per year (Trombetti et al., 2016). Lower limb muscle strength is a crucial determinant of ambulatory capacity (Yeung et al., 2019). Impairments in sit-to-stand ability and gait speed due to sarcopenia may increase the risk of falls during ambulation in older adults (Rodrigues et al., 2022). The ability to perform sit-to-stand transfers is important for maintaining independent living and reducing fall-related fractures, as well as preserving normal gait function in this population (Warzecha et al., 2020). Gait asymmetry has been reported in older adults, tends to increase with advancing age, and has been associated with impaired mobility, balance deficits, and an increased risk of falls (Laroche et al., 2012).

Sarcopenia-related declines in lower-limb muscle strength, sit-to-stand ability, and gait performance may disrupt gait rhythmicity and increase inter-stride variability, both of which are recognized modifiable risk factors for falls in older adults (Vikberg et al., 2019; Zhao et al., 2022). Non-pharmacological interventions, particularly exercise and nutritional approaches, are recognized as effective management strategies for sarcopenia (Lu et al., 2021). Exercise interventions demonstrate significant efficacy in improving muscle strength and physical function among older adults with sarcopenia (Stoever et al., 2018; Yang et al., 2022), with emerging evidence supporting resistance training as a promising non-pharmacological intervention (Keating et al., 2021; Zhao et al., 2022).

Among different resistance-training modalities, elastic band-based resistance training may be a practical and feasible option for older adults with sarcopenia. Elastic bands are inexpensive, portable, generally safe, and easy to implement in community or home-based settings without requiring specialized equipment or large training spaces (Martins et al., 2015; Liao et al., 2017). Compared with machine-based resistance training, elastic bands offer greater practical flexibility because exercise intensity can be progressively adjusted by changing band stiffness, elongation length, grip position, range of motion, exercise tempo, or repetition number, making this modality suitable for older adults with heterogeneous strength levels and functional limitations (Lopes et al., 2019). Previous randomized trials and meta-analyses have reported that elastic band-based resistance training can improve muscle strength, body composition, physical function, balance, gait speed, and lower-limb functional performance in older adults, including those with sarcopenia or sarcopenic obesity (Liao et al., 2017; Liao et al., 2018; Valdés-Badilla et al., 2023; Meng et al., 2025). More recently, a systematic review and meta-analysis further confirmed that elastic band resistance training significantly improves lower-limb strength, chair-stand performance, functional mobility, and balance in older adults (Meng et al., 2025). However, most existing studies have focused primarily on global strength, body composition, or general physical performance, whereas fewer studies have examined whether elastic band-based resistance training can simultaneously improve task-specific sit-to-stand performance, lower-limb muscle activation, balance function, and gait symmetry in older adults with sarcopenia.

Therefore, this study evaluated pre–post changes in lower-limb EMG-derived activation indices, sit-to-stand transfer ability, balance function, and gait symmetry after a 12-week elastic band-based resistance training program in older adults with sarcopenia. We hypothesized that post-intervention assessments would show: (1) a reduction in the time required to complete the FTSST; (2) higher surface electromyographic activation indices, as reflected by RMS and IEMG values, in key lower-limb muscles; (3) higher BBS scores; and (4) lower gait symmetry indices, including step length, stance phase, swing phase, and contact time symmetry indices.

## 2 Materials and methods

### 2.1 Study design and participants

This study used a pragmatic single-arm pre–post design to evaluate changes in sit-to-stand performance, EMG-derived activation indices, balance function, and gait symmetry after a 12-week elastic band training program in older adults with sarcopenia. Because the program was implemented as a community-based exercise initiative, all eligible participants were scheduled to receive the training; a concurrent untreated control group was therefore not included. Initially, 120 community-dwelling older adults with sarcopenia were recruited. Of these, 102 participants completed the 12-week intervention and post-intervention assessments and were included in the final analysis. All pre–post statistical analyses were conducted using data from these 102 participants. The final sample consisted of 48 men and 54 women, with a mean age of 67.32 ± 2.90 years and an age range of 61.5–73.1 years.

*A priori* power analysis was conducted using G*Power 3.1 for the difference between two dependent means (matched pairs), based on a pilot-derived standardized paired effect size of d_z_ = 0.394, a two-tailed α = 0.05, and power (1 − β) = 0.95. The pilot study included 12 participants, with a mean within-participant FTSST change of −0.90 s and a standard deviation of 2.284 s for the paired differences; therefore, d_z_ was calculated as |−0.90/2.284| = 0.394. The analysis indicated that a minimum of 86 participants with complete pre–post data was required. Therefore, the final sample of 102 participants exceeded the required minimum sample size. The initial recruitment target of 120 participants was set to allow for potential attrition.

Before commencing the experiment, all participants received detailed information about the study objectives, procedures, potential benefits, and possible risks. Written informed consent was obtained from each participant before enrollment. This study was conducted in accordance with the ethical principles of the Declaration of Helsinki and was approved by the Academic Ethics Committee of Jilin Sport University (Approval No. 2025009). This study was not prospectively registered in a clinical trial registry.

#### 2.1.1 Diagnostic criteria

Sarcopenia was diagnosed according to the Guideline for diagnosis and treatment of sarcopenia in China (2024 edition) (Geriatrics Branch of the Chinese Medical Association and National Clinical Research Center for Geriatric Disorders (Xiangya Hospital), 2025). Participants were diagnosed with sarcopenia if they concurrently presented with low physical performance, low muscle strength, and low appendicular skeletal muscle mass index. Specifically, the diagnostic criteria were a usual gait speed of less than 1.0 m/s, handgrip strength of less than 28 kg for men or less than 18 kg for women, and appendicular skeletal muscle mass index measured by bioelectrical impedance analysis of less than 7.0 kg/m² for men or less than 5.7 kg/m² for women.

#### 2.1.2 Eligibility criteria

Participants were eligible for inclusion if they met the diagnostic criteria for sarcopenia described above, were aged 60 years or older, were able to walk independently, were able to understand and complete the required functional assessments, had a Mini-Mental State Examination score greater than 26, and voluntarily provided written informed consent (Han et al., 2018; Geriatrics Branch of the Chinese Medical Association and National Clinical Research Center for Geriatric Disorders (Xiangya Hospital), 2025). Participants were also required to pass a physical health examination confirming that they were able to safely participate in the exercise intervention.

Participants were excluded if they had severe cardiovascular disease classified as New York Heart Association class III–IV, severe neurological disease, severe musculoskeletal disorders that limited exercise participation, recent lower-limb fracture or surgery, malignant tumors, severe psychiatric disorders affecting independent daily functioning, participation in other exercise intervention studies within the preceding three months, severe osteoporosis defined by a bone mineral density T-score below −2.5, or severe uncontrolled metabolic conditions that could compromise exercise safety or prevent valid completion of the functional assessments (Pagotto and Silveira, 2014; Sánchez-Rodríguez et al., 2016; Shimizu et al., 2022). Diabetes, obesity, or metabolic syndrome was not considered an exclusion criterion in itself unless the condition was uncontrolled or was judged during the physical health examination to interfere with safe participation or valid completion of the assessments. Participants were also excluded if they had acute illness or other conditions that could prevent safe completion of the intervention or functional assessments.

On each assessment day, participants were screened before testing for acute infection, fever, unusual fatigue, acute musculoskeletal pain, dizziness, or other symptoms that could influence physical performance. Testing was postponed if any of these conditions were present. Participants were instructed to avoid strenuous physical activity, alcohol, caffeine, and non-essential pain medication before the assessment. They were also asked to report any medication or drug use that might affect neuromuscular function, balance, gait, or sit-to-stand performance. These procedures were used to minimize the potential influence of acute health status, fatigue, stimulant intake, or medication use on the functional and gait assessments.

### 2.2 Intervention

Participants completed a 12-week supervised elastic band-based resistance training program, with three sessions per week and each session lasting approximately 60 min. Each session consisted of three parts: a 10-min warm-up, 40–45 min of elastic band-based resistance and balance exercises, and a 5–10 min cool-down. The intervention was delivered as supervised small-group classes rather than individual appointments. Each class included approximately 10–12 participants and was supervised by one lead instructor and one assistant, corresponding to an instructor-to-participant ratio of approximately 1:5 to 1:6. At the beginning of each session, the instructor demonstrated each exercise and emphasized key movement points and safety precautions. During the main training component, the instructors circulated among participants to correct movement technique, monitor fatigue and discomfort, prevent compensatory movements, and adjust the training load according to participant tolerance and movement quality. After each session, participants were asked to report their general training experience, including whether the exercises felt too easy, appropriate, or too difficult, and whether they experienced discomfort or pain.

The intervention used elastic bands with four progressive resistance levels: yellow, red, green, and blue, corresponding to low, moderate, high, and very high resistance, respectively. According to the manufacturer’s resistance chart at 100% elongation, these bands provide approximately 0.5–1.3 lbs, 1.3–2.6 lbs, 2.6–4.5 lbs, and 4.5–7.2 lbs of resistance, respectively. Three band lengths were available, including 8 inches (20 cm), 12 inches (30 cm), and 18 inches (46 cm), to accommodate different exercise types, limb lengths, movement ranges, and participant strength levels.

Yellow bands were used as the initial resistance level during the early familiarization phase because they provided relatively low external resistance and allowed older adults with sarcopenia to learn the exercise movements safely and with proper technique (Huang et al., 2017; Valdés-Badilla et al., 2023). However, the yellow band was not used as a fixed resistance for all participants throughout the intervention. Because a formal Borg RPE scale, 0–10 RPE scale, or visual analog scale was not recorded, training intensity was individualized according to participant-reported training tolerance, post-session feedback, movement quality, and the ability to complete the prescribed repetitions.

Progression was determined using a stepwise approach. When participants could complete the lower end of the target repetition range with correct technique and without excessive fatigue or discomfort, the number of repetitions was first increased within the prescribed range. When participants could complete the upper end of the target repetition range with good movement control and reported that the exercise felt manageable, resistance was progressed by increasing band elongation, shortening the grip length, selecting a shorter band length when appropriate, or progressing to a higher-resistance band color. If participants showed poor movement control, pain, excessive fatigue, or inability to complete the target repetitions, the resistance level or repetition number was reduced.

The exercise protocol targeted major upper- and lower-limb muscle groups, with an emphasis on functional movements related to sit-to-stand transfer, postural control, and gait. The program included eight exercises: (1) Sideways Bow Drawing, (2) Chest Push-Out, (3) Lateral Shoulder Abduction, (4) Standing Hip and Knee Flexion with Band Resistance, (5) Seated Knee Extension, (6) Standing Hip Extension, (7) Single-Leg Stand, and (8) Standing Heel Raises. Each exercise was performed for 2 sets of 10–15 repetitions, except for single-leg standing, which was performed for 2 sets of 2–2.5 min per leg, and standing heel raises, which were performed for 2 sets of 20 repetitions. For unilateral exercises, completing both the left and right sides was considered one set. Exercises 7 and 8 were body-weight balance and calf-raise exercises and did not require elastic bands. Detailed descriptions of the training protocol, including target muscles, resistance adjustment, training dose, progression criteria, and functional relevance, are provided in **Supplementary Table S1**.

### 2.3 Electromyography

Surface electromyography (sEMG) was recorded using the BTS FreeEMG 1000 system (BTS Bioengineering, Italy), a wireless surface EMG system, during the sit-to-stand task. Signals were collected bilaterally from the rectus femoris (RF), tibialis anterior (TA), and medial gastrocnemius (Gas) muscles. A bipolar surface electrode configuration was used. Before electrode placement, the skin was shaved when necessary, gently abraded, and cleaned with alcohol to reduce skin impedance. Electrodes were placed according to SENIAM recommendations and aligned parallel to the presumed direction of the muscle fibers (Hermens et al., 2000).

For the RF, electrodes were placed at 50% of the line from the anterior superior iliac spine to the superior border of the patella. For the TA, electrodes were placed at one-third of the line between the tip of the fibula and the tip of the medial malleolus. For the medial gastrocnemius, electrodes were placed over the most prominent bulge of the muscle belly and aligned in the longitudinal direction of the leg. All electrode placements were performed by the same trained assessor, and the placement sites were marked to improve consistency between baseline and post-intervention assessments.

The sEMG signal was sampled at 1000 Hz, band-pass filtered at 20–450 Hz, and notch-filtered at 50 Hz to reduce movement artifacts and power-line interference. Signals were full-wave rectified. Windowed RMS values were calculated using a 20-ms window. One formal sit-to-stand trial was recorded for EMG analysis at each assessment. For this trial, the analysis epoch began with the standardized auditory start command and ended when the participant reached a fully upright standing position. The root mean square (RMS) and integrated EMG (IEMG) were calculated only within this predefined cue-to-upright-standing epoch. EMG and video were recorded synchronously. The end of the analysis epoch was identified by visual inspection of the synchronized video as the time at which the participant attained a fully upright standing position; the corresponding time point was used to delimit the EMG signal. The same procedure was used at baseline and post-intervention assessments.

A resting EMG baseline was recorded while the participant was seated quietly during the preparation period. RMS_task_ denotes the RMS calculated over the entire cue-to-upright-standing epoch, RMS_rest_ denotes the RMS of the seated resting baseline, and RMS_peak_ denotes the maximum 20-ms windowed RMS within that same task epoch. The task-derived reference was obtained separately for each participant, muscle, and assessment. The calculation was defined as: Task-normalized RMS (%) = (RMS_task_ − RMS_rest_)/(RMS_peak_ − RMS_rest_) × 100. Because the participants were older adults with sarcopenia, a separate standardized maximum voluntary isometric contraction (MVIC) test was not performed because it could impose unnecessary physical demands and reduce testing feasibility. Accordingly, the RMS values were described as task-normalized RMS rather than percentages of MVIC.

IEMG was calculated from the processed sEMG signal over the same cue-to-upright-standing epoch and was reported in its original amplitude-related unit (μV·s). IEMG was not normalized to MVIC or to the task-related RMS reference.

### 2.4 Familiarization with testing procedures

To minimize potential learning effects, all participants received standardized verbal instructions and demonstrations before each functional and gait assessment. Before formal EMG recording, participants practiced responding to the auditory start cue and standing up fully. One formal sit-to-stand trial was then recorded at each assessment. The assessor confirmed that each participant understood the command and the required movement before formal data collection. Participants then completed one familiarization trial for the Five Times Sit-to-Stand Test, Timed Up and Go Test, and sit-to-stand transfer task. Familiarization and adaptation practice were not included in the statistical analysis.

For the Five Times Sit-to-Stand Test and Timed Up and Go Test, participants then completed two formal trials, with a 1–2 min seated rest interval between trials, and the shorter completion time of the two formal trials was used for analysis. For the sit-to-stand transfer task, participants completed one familiarization trial followed by two formal trials, and the shorter transfer time was recorded as the final outcome measure.

For gait assessment, participants were allowed to walk on the DIERS pedogait treadmill for approximately 5 min to adapt to the testing environment and treadmill speed before formal data recording. Formal gait recordings were started only after participants achieved a stable walking pattern. The same familiarization procedures, standardized instructions, testing environment, and assessor supervision were used at baseline and post-intervention assessments.

### 2.5 Five times sit-to-stand test

The primary outcome reported was the change in FTSST completion time, measured in seconds, from baseline to the end of the 12-week program. A prospective hierarchy of primary and secondary outcomes was not explicitly documented. The selection of FTSST reflected the study’s focus on lower-limb functional performance, particularly repeated sit-to-stand ability. Previous research in community-dwelling older adults has shown that poorer FTSST performance predicts subsequent disability, supporting its relevance to functional assessment in this population (Makizako et al., 2017). Participants were seated on a 48-cm-high armless chair, which was placed against a wall to prevent movement. They were instructed to cross their arms over their chest and keep both feet flat on the floor. After the familiarization procedure described above, participants were instructed to stand up fully and sit down five times as quickly as possible following the verbal cue “start.” A stopwatch was used to record the total time, in seconds, from the initial command to the completion of the fifth stand-to-sit cycle. Each participant completed two formal FTSST trials, with a 1–2 min seated rest interval between trials to minimize fatigue. The shorter completion time of the two formal trials was used for subsequent statistical analysis (Makizako et al., 2017).

### 2.6 Timed Up and Go Test

The Timed Up and Go Test (TUGT) was employed to assess functional mobility, dynamic balance, and gait performance. Participants began seated in a standard-height, armless chair positioned at the starting line, with a clearly marked 3-m distance ahead. Upon the verbal command “start,” participants were instructed to rise from the chair, walk at a comfortable and safe pace to the 3-m line, turn around, walk back, and return to a seated position. The total time, in seconds, was recorded using a stopwatch, starting from the moment the participant’s buttocks left the seat until they returned to contact the chair. After the familiarization procedure described above, each participant completed two formal TUGT trials, with a 1–2 min seated rest interval between trials. The shorter completion time of the two formal trials was used for subsequent statistical analysis. Shorter completion times were interpreted as indicative of better functional mobility, dynamic balance, and overall mobility (Benavent-Caballer et al., 2016; Medina-Mirapeix et al., 2018).

### 2.7 Sit-to-stand transfer time

A sit-to-stand-and-step task adapted from the lie-to-sit-to-stand-to-walk transfer test (Kacmaz et al., 2024) was used in this study. Sit-to-stand transfer time was defined in this study as the interval from the auditory start cue to clearance of the trailing limb from the ground during a sit-to-stand-and-step task. Participants were seated in a standardized position at the designated starting point. Upon receiving an auditory start cue, participants were instructed to stand up from the chair and initiate a forward step as naturally and safely as possible. Timing began at the delivery of the auditory cue and ended when the trailing limb cleared the ground. After the familiarization procedure described above, each participant completed two formal trials, with a 1–2 min seated rest interval between trials. The shorter transfer time of the two formal trials was recorded as the final outcome measure (Kacmaz et al., 2024).

### 2.8 Berg Balance Scale

The Berg Balance Scale (BBS) was used to assess balance performance in older adults (Berg et al., 1992). The BBS was used as a secondary outcome to evaluate pre–post changes in balance performance during the study period.

### 2.9 Gait symmetry parameters

Gait symmetry indices, including step length, stance phase, swing phase, and contact time symmetry indices, were assessed to evaluate pre–post changes in bilateral coordination during ambulation. The DIERS pedogait system (DIERS International GmbH, Schlangenbad, Germany) was used to assess gait parameters and plantar pressure. Participants were instructed to walk barefoot on the treadmill at a self-selected comfortable and safe speed. After approximately 5 min of treadmill familiarization, the speed was determined for each participant according to their subjective perception of comfort and safety under assessor supervision. Once a stable walking pattern had been achieved, the participant-specific target velocity was displayed and maintained during three consecutive 30-second kinematic data recordings. The same participant-specific target speed was used at baseline and post-intervention assessments. The mean values of the three recordings served as the definitive outcome measures. The same speed-selection procedure, testing environment, standardized instructions, and assessor supervision were used at both assessments. The following test indices were documented:

Step Length (cm): This refers to the distance measured from the trailing point of one foot to the trailing point of the opposite foot.

Stance phase (%): the percentage of the gait cycle during which the foot of a given limb remained in contact with the treadmill. This variable was recorded separately for the left and right limbs.

Swing phase (%): the percentage of the gait cycle during which the foot of a given limb was not in contact with the treadmill. This variable was recorded separately for the left and right limbs.

Contact time (s): the duration for which one foot remained in contact with the treadmill during the stance phase of a gait cycle. This variable was recorded separately for the left and right feet and was expressed in seconds.

For each gait variable, values were recorded separately for the left and right limbs and were then used to calculate the corresponding symmetry index. No fixed reference side was used for all participants, and the calculation was not based on limb dominance. The symmetry index (SI) was calculated as follows:


$$SI=\frac{\left|X_{L}-X_{R}\right|}{0.5\times (X_{L}+X_{R})}\times 100\%$$

where *X_L_* denotes the value of the gait variable for the left limb, and *X_R_* denotes the corresponding value for the right limb. An SI of 0 indicates perfect bilateral symmetry, whereas larger SI values indicate greater inter-limb asymmetry. Although the original gait variables were expressed in different units, including cm, %, and s, the calculated SI values were dimensionless and expressed as percentages. SI values were used to quantify inter-limb asymmetry (VanZant et al., 2001; Wafai et al., 2013; Błażkiewicz et al., 2014), with lower values indicating greater bilateral symmetry.

### 2.10 Statistical analysis

Statistical analyses were performed using R software (version 4.5.3; R Foundation for Statistical Computing, Vienna, Austria). *Post hoc* sensitivity analyses were conducted using Python (version 3.13.5) and SciPy (version 1.16.2). Continuous variables are presented as mean ± standard deviation (SD), and categorical variables as counts. The analyses included the 102 participants who completed both baseline and post-intervention assessments. Participants without post-intervention assessments were excluded from the pre–post analyses. Baseline characteristics were summarized for the overall sample and by sex. For each outcome, the mean change was calculated as the post-intervention value minus the pre-intervention value.

The normality of paired differences was assessed using the Shapiro–Wilk test and diagnostic plots. Paired-samples t-tests were used to compare pre- and post-intervention EMG outcomes, FTSST, TUGT, sit-to-stand transfer time, and contact-time symmetry index. Wilcoxon signed-rank tests were used for BBS scores and step-length, stance-phase, and swing-phase symmetry indices. As a *post hoc* sensitivity analysis, Wilcoxon signed-rank tests were additionally performed for all 12 EMG comparisons, with Holm adjustment applied within the EMG outcome family.

FTSST was reported as the primary outcome, without a claim of documented prospective specification, and its P value was not adjusted for multiplicity. Holm adjustment was applied separately within the secondary functional outcome family (TUGT and sit-to-stand transfer time), the 12 EMG comparisons, and the four gait symmetry outcomes. The BBS comparison was reported without multiplicity adjustment. These adjustments control multiplicity within the specified families rather than across all study outcomes. In the absence of an explicitly documented prospective outcome hierarchy, the analyses should be interpreted as exploratory.

For outcomes analyzed using paired-samples t-tests as the primary inferential test, standardized within-participant changes were reported as Cohen’s *d*_z_, calculated as the mean post-intervention minus pre-intervention difference divided by the standard deviation of the paired differences (*d*_z_ = mean(Post − Pre)/SD_difference_). The 95% confidence intervals for mean changes were estimated using 5,000 paired bootstrap resamples with a fixed random seed. Negative *d*_z_ values indicate reductions, whereas positive *d*_z_ values indicated increases; the direction of improvement depended on the outcome measure. For outcomes analyzed using Wilcoxon signed-rank tests, the effect-size reporting is described separately.

For outcomes analyzed using Wilcoxon signed-rank tests, effect sizes were reported as matched-pairs rank-biserial correlations (*r_rb_*). The *r_rb_* values were calculated from the participant-level post-minus-pre differences after excluding zero differences. Positive values indicate predominantly positive changes, whereas negative values indicate predominantly negative changes.

## 3 Results

### 3.1 Characteristics of participants

Of the 120 participants initially recruited, 102 individuals completed the 12-week intervention and post-intervention assessments and were included in the complete-case analysis. Among these completers, mean attendance was 33.16 ± 1.89 sessions (92.10%; range, 80.56–100.0%), and all 102 participants attended at least 80% of the 36 scheduled sessions, corresponding to at least 29 sessions. The remaining 18 participants attended 12.56 ± 2.77 sessions on average (34.88%; range, 22.22–47.22%), corresponding to 8–17 sessions, and did not complete the intervention and post-intervention assessment. The overall attendance was 30.07 ± 7.66 sessions (83.52%; range, 22.22–100.0%). Reasons for non-completion were personal reasons (n = 6), illness causing mobility difficulties (n = 5), and loss to follow-up (n = 7). Some participants reported muscle soreness after exercise. No exercise-related injuries were reported during the intervention.

Baseline characteristics of the participants are summarized in **Table 1**. Men were generally taller and heavier than women and had higher appendicular skeletal muscle index and handgrip strength, whereas body mass index, usual gait speed, and Berg Balance Scale scores were comparable between sexes. All participants met the predefined diagnostic criteria for sarcopenia at baseline.

**Table 1:** Baseline characteristics of the participants.

Characteristic	Overall (n = 102)	Male (n = 48)	Female (n = 54)
Age (years)	67.32 ± 2.90	68.13 ± 2.76	66.60 ± 2.86
Height (cm)	159.59 ± 5.84	164.92 ± 2.90	154.86 ± 3.00
Weight (kg)	60.93 ± 5.65	65.21 ± 4.56	57.12 ± 3.32
BMI (kg/m²)	23.89 ± 1.39	24.00 ± 1.89	23.80 ± 0.71
ASMI (kg/m²)	5.68 ± 0.81	6.37 ± 0.59	5.07 ± 0.37
Handgrip strength (kg)	19.51 ± 4.74	24.08 ± 2.40	15.46 ± 1.45
Gait speed (m/s)	0.6912 ± 0.1425	0.6869 ± 0.1428	0.6950 ± 0.1435
Berg Balance Scale (score)	41.37 ± 1.99	41.35 ± 2.18	41.39 ± 1.83

Data are presented as mean ± standard deviation (SD). Baseline characteristics are summarized for all participants and stratified by gender. BMI, body mass index; ASMI, appendicular skeletal muscle index.

### 3.2 Electromyography analysis

To evaluate our second hypothesis, task-specific neuromuscular activation during the sit-to-stand task was evaluated using surface electromyography (sEMG), including root mean square (RMS) and integrated electromyography (IEMG) of the bilateral rectus femoris (RF), tibialis anterior (TA), and gastrocnemius (Gas) muscles.

Following the 12-week elastic band resistance training program, Task-normalized RMS values decreased significantly in all examined muscles, including the bilateral RF, TA, and Gas muscles (all Holm-adjusted P < 0.05). For IEMG, significant increases were observed in the bilateral RF and TA muscles, whereas no significant changes were detected in the bilateral Gas muscles after Holm adjustment (all adjusted P > 0.05).

Overall, the intervention was associated with reductions in task-normalized RMS values and muscle-specific changes in IEMG during the sit-to-stand task, particularly in the rectus femoris and tibialis anterior muscles. Detailed pre- and post-intervention comparisons are presented in **Table 2** and **Figure 1**. In *post hoc* sensitivity analyses using Wilcoxon signed-rank tests with the same 12-comparison Holm adjustment, the pattern of statistically significant and nonsignificant EMG comparisons was unchanged.

**Table 2:** Changes in lower-limb EMG variables during the sit-to-stand task before and after the 12-week elastic band resistance training program.

Outcome	Pre-intervention (Mean ± SD)	Post-intervention (Mean ± SD)	Mean difference (95% CI)	Statistic	*P* value	Cohen’s *d*_z_
Task-normalized RMS (%)						
LRF	30.51 ± 13.37	26.69 ± 12.55	−3.82 (−4.75, −2.90)	t(101) = −8.19	<0.001	−0.81
RRF	28.90 ± 9.63	25.09 ± 9.02	−3.81 (−4.50, −3.12)	t(101) = −10.94	<0.001	−1.08
LGM	13.86 ± 9.01	12.87 ± 9.15	−0.99 (−1.72, −0.26)	t(101) = −2.68	0.028	−0.27
RGM	15.98 ± 10.64	13.83 ± 9.06	−2.14 (−3.00, −1.29)	t(101) = −4.95	<0.001	−0.49
LTA	34.97 ± 14.39	30.98 ± 15.48	−3.99 (−5.23, −2.75)	t(101) = −6.37	<0.001	−0.63
RTA	34.97 ± 16.78	30.76 ± 16.61	−4.21 (−5.47, −2.95)	t(101) = −6.65	<0.001	−0.66
IEMG						
LRF	92.29 ± 42.39	101.76 ± 42.08	9.48 (3.23, 15.82)	*t*(101) = 2.97	0.018	0.29
RRF	94.08 ± 43.83	102.50 ± 45.00	8.42 (2.49, 14.56)	*t*(101) = 2.75	0.028	0.27
LGM	86.66 ± 37.48	89.02 ± 39.01	2.35 (−2.93, 7.49)	*t*(101) = 0.89	0.376	0.09
RGM	81.53 ± 38.78	85.46 ± 37.31	3.93 (−1.89, 9.71)	*t*(101) = 1.37	0.344	0.14
LTA	162.19 ± 68.94	188.16 ± 77.62	25.97 (15.55, 36.38)	*t*(101) = 4.83	<0.001	0.48
RTA	156.28 ± 69.37	179.00 ± 76.00	22.72 (12.46, 33.21)	*t*(101) = 4.29	<0.001	0.43

Data are presented as mean ± standard deviation (SD). Mean differences were calculated as post-intervention minus pre-intervention values. Primary analyses of all EMG variables were performed using paired-samples t-tests. *Post hoc* Wilcoxon signed-rank sensitivity analyses were conducted for all 12 EMG comparisons. The reported P values were adjusted using the Holm procedure within the EMG outcome family. Effect sizes are presented as Cohen’s *d*z, calculated as the mean post-intervention minus pre-intervention difference divided by the standard deviation of the paired differences. LRF, left rectus femoris; RRF, right rectus femoris; LGM, left gastrocnemius; RGM, right gastrocnemius; LTA, left tibialis anterior; RTA, right tibialis anterior; RMS, root mean square; IEMG, integrated electromyography. Task-normalized RMS values were calculated after correction for the resting baseline using the predefined cue-to-upright-standing epoch; no independent maximum voluntary isometric contraction (MVIC) normalization was performed.

**Figure 1:**
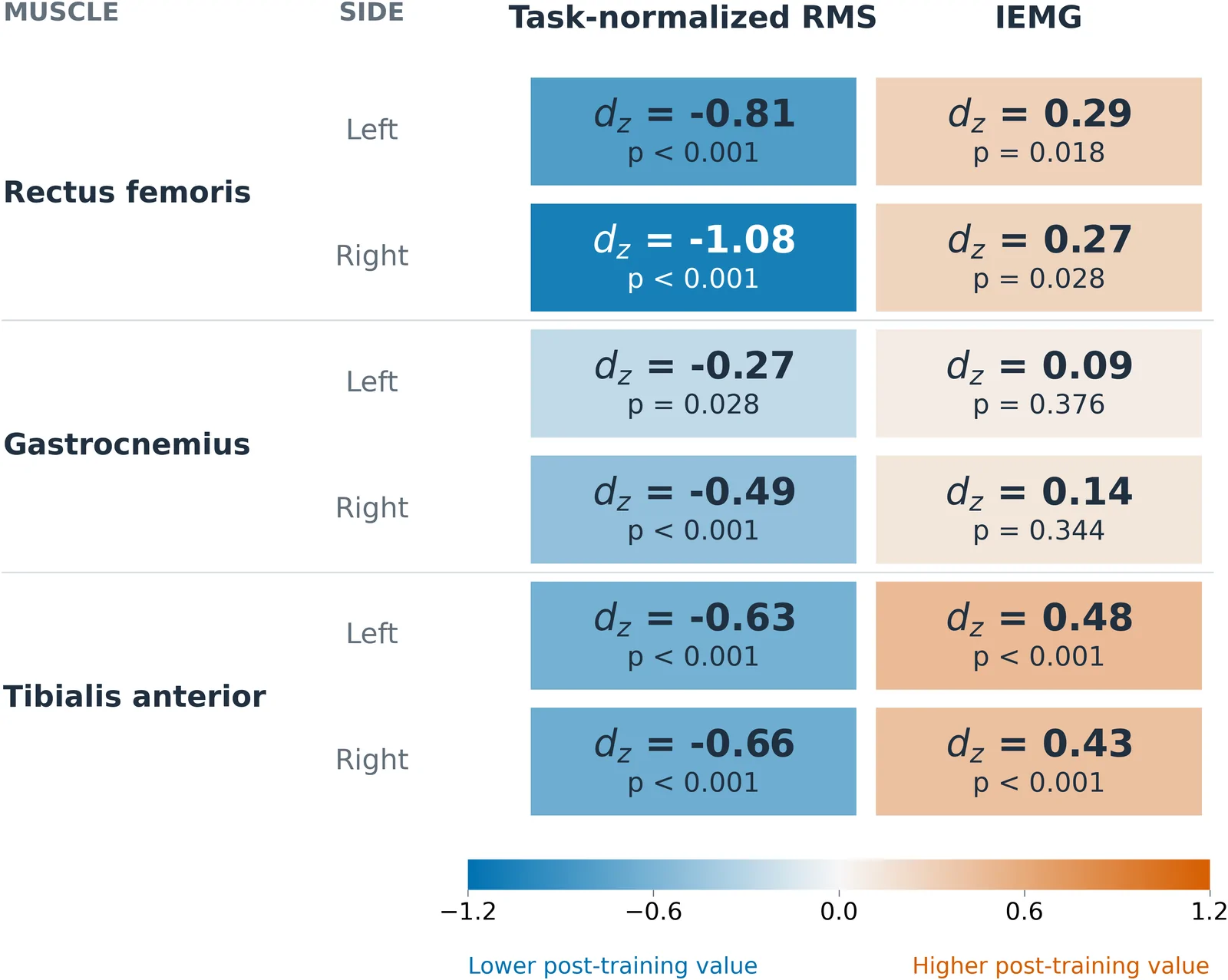
Standardized pre–post changes in task-related electromyographic indices among 102 completers. Each cell shows Cohen’s *d*_z_ and the Holm-adjusted *P* value for one muscle–outcome pair. Cohen’s *d*_z_ was calculated as the mean post-intervention value minus the mean pre-intervention value, divided by the standard deviation of the paired differences. Blue indicates lower post-intervention values (negative *d*_z_), whereas orange indicates higher values (positive *d*_z_); the color scale is centered at zero. *P* values were obtained from paired-samples *t*-tests and adjusted across the 12 EMG comparisons. RMS, root mean square, expressed as a task-normalized percentage; IEMG, integrated electromyography, expressed in μV·s. Colors represent standardized effect sizes rather than raw EMG amplitudes.

### 3.3 Balance function

As shown in **Table 3** and **Figure 2**, the Berg Balance Scale (BBS) score increased significantly from 41.37 ± 1.99 at baseline to 46.96 ± 1.88 after the 12-week elastic band resistance training program (Wilcoxon signed-rank test, P < 0.001; *r_rb_* = 1.00). The mean change was 5.59 points (95% CI, 5.30–5.89), indicating improved balance performance following the intervention.

**Table 3:** Comparison of Berg Balance Scale scores before and after the 12-week elastic band resistance training program.

Outcome	Pre-intervention (Mean ± SD)	Post-intervention (Mean ± SD)	Mean difference (95% CI)	Statistic	P value	*r_rb_*
Berg Balance Scale (score)	41.37 ± 1.99	46.96 ± 1.88	5.59 (95% CI: 5.30, 5.89)	V = 5253	<0.001	1.00

Data are presented as mean ± standard deviation (SD). BBS scores were analyzed using the Wilcoxon signed-rank test. The effect size is presented as the matched-pairs rank-biserial correlation (*r_rb_*), calculated from the participant-level post-minus-pre differences after excluding zero differences. Positive values indicate predominantly higher post-intervention scores.

**Figure 2:**
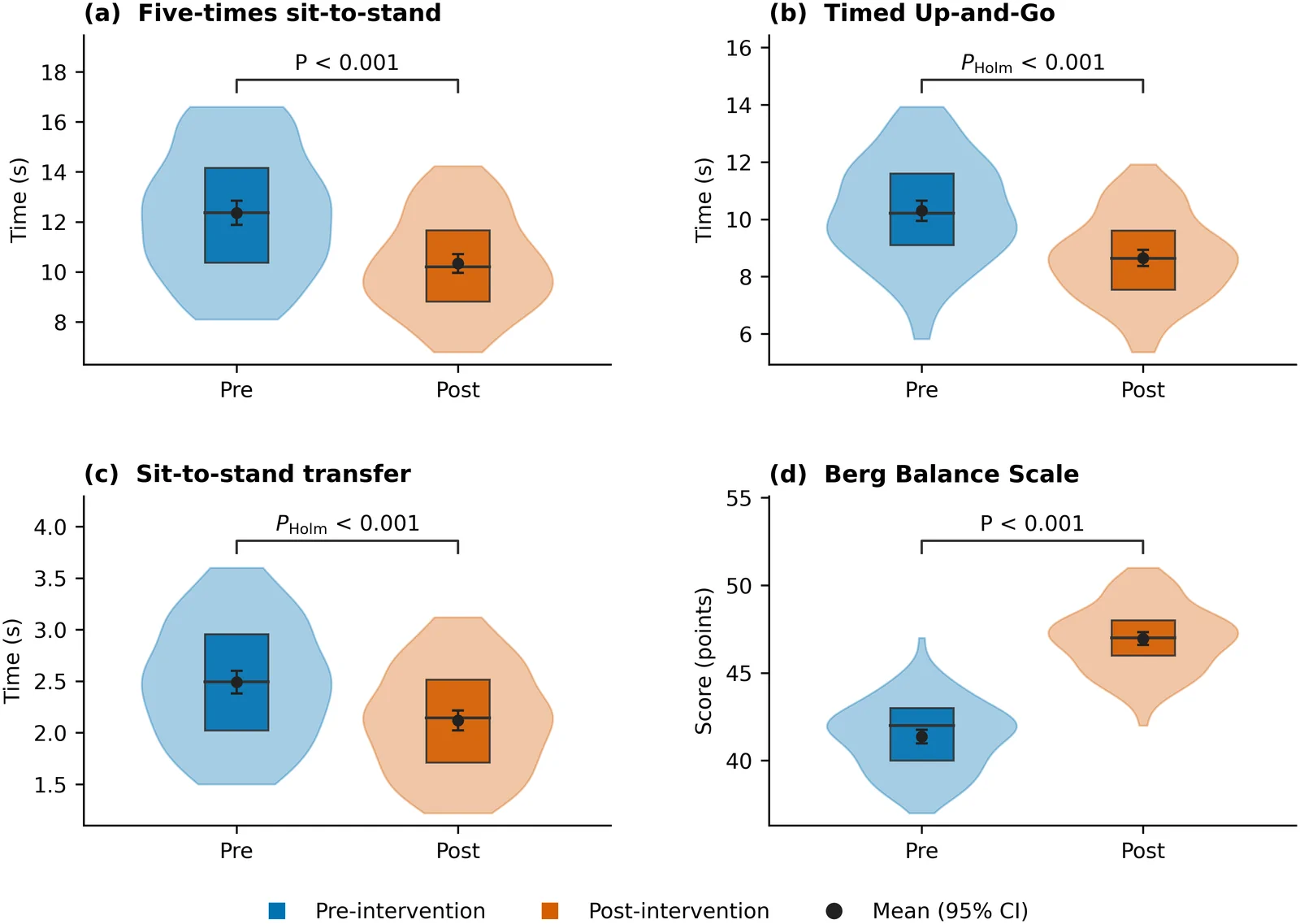
Distributions of functional performance and balance before and after the 12-week combined exercise program among 102 completers. Panels show **(a)** the Five Times Sit-to-Stand Test (FTSST), **(b)** the Timed Up and Go Test (TUGT), **(c)** sit-to-stand transfer time, and **(d)** the Berg Balance Scale (BBS). Blue and orange indicate pre- and post-intervention assessments, respectively. Violin shapes represent estimated distributions, boxes represent the interquartile range, horizontal lines indicate medians, and black points indicate means. Error bars represent approximate 95% confidence intervals for the mean, calculated as mean ± 1.96 × standard error; these intervals do not represent paired mean changes. Shorter completion times and higher BBS scores indicate better performance. Brackets indicate within-participant pre–post comparisons. FTSST, TUGT, and sit-to-stand transfer time were analyzed using paired-samples *t*-tests, whereas BBS scores were analyzed using the Wilcoxon signed-rank test. Holm adjustment was applied to TUGT and sit-to-stand transfer time; no multiplicity adjustment was applied to FTSST or BBS. All displayed comparisons yielded *P* < 0.001.

### 3.4 Lower-limb muscle function

FTSST, TUGT, and sit-to-stand transfer time were assessed before and after the intervention to evaluate changes in lower-limb functional performance. As shown in **Table 4** and **Figure 2**, significant improvements were observed in all three functional performance measures following the 12-week elastic band resistance training program.

**Table 4:** Pre- and post-intervention comparison of FTSST, TUGT, and sit-to-stand transfer time.

Outcome	Pre-intervention (Mean ± SD)	Post-intervention (Mean ± SD)	Mean difference (95% CI)	Statistic	*P* value	Cohen’s *d*_z_
Five Times Sit-to-Stand Test (s)	12.36 ± 2.48	10.34 ± 1.91	−2.02 (−2.17, −1.87)	*t*(101) = −26.73	<0.001	−2.65
Timed Up and Go Test (s)	10.30 ± 1.80	8.65 ± 1.46	−1.65 (−1.85, −1.45)	*t*(101) = −15.58	<0.001	−1.54
Sit-to-Stand Transfer Time (s)	2.49 ± 0.57	2.12 ± 0.50	−0.37 (−0.39, −0.35)	*t*(101) = −36.83	<0.001	−3.65

Data are presented as mean ± standard deviation (SD). Mean differences were calculated as post-intervention minus pre-intervention values. FTSST, TUGT, and sit-to-stand transfer time were analyzed using paired-samples t-tests. P values for TUGT and sit-to-stand transfer time were adjusted using the Holm procedure within the specified functional secondary outcome family. Effect sizes are presented as Cohen’s *d*z, calculated as the mean post-intervention minus pre-intervention difference divided by the standard deviation of the paired differences.

Specifically, FTSST completion time decreased significantly from 12.36 ± 2.48 s at baseline to 10.34 ± 1.91 s after the intervention (mean difference: −2.02 s, 95% CI: −2.17 to −1.87; t(101) = −26.73, P < 0.001; Cohen’s *d*z = −2.65). Similarly, TUGT time decreased from 10.30 ± 1.80 s to 8.65 ± 1.46 s (mean difference: −1.65 s, 95% CI: −1.85 to −1.45; t(101) = −15.58, P < 0.001; Cohen’s *d*z = −1.54). Sit-to-stand transfer time also decreased significantly, from 2.49 ± 0.57 s to 2.12 ± 0.50 s (mean difference: −0.37 s, 95% CI: −0.39 to −0.35; t(101) = −36.83, P < 0.001; Cohen’s *d*z = −3.65).

Overall, these findings indicate that the intervention was associated with better lower-limb functional performance and functional mobility in older adults with sarcopenia.

### 3.5 Gait symmetry

Consistent with the fourth hypothesis, significant improvements were observed in gait symmetry following the 12-week elastic band resistance training program. As shown in **Table 5** and **Figure 3**, all four gait symmetry indices demonstrated significant reductions in inter-limb asymmetry after the intervention.

**Table 5:** Changes in gait symmetry indices following the 12-week elastic band resistance training program.

Outcome	Pre-intervention (Mean ± SD)	Post-intervention (Mean ± SD)	Mean difference (95% CI)	Statistic	*P* value	Effect size
Step length symmetry index (%)	15.66 ± 2.86	10.91 ± 4.82	−4.75 (−5.32, −4.13)	*V* = 161	<0.001	−0.94
Stance phase symmetry index (%)	3.88 ± 2.84	2.35 ± 1.95	−1.53 (−1.80, −1.27)	*V* = 0	<0.001	−1.00
Swing phase symmetry index (%)	8.50 ± 6.31	4.12 ± 3.57	−4.38 (−4.98, −3.79)	*V* = 0	<0.001	−1.00
Contact time symmetry index (%)	8.09 ± 1.55	6.01 ± 2.03	−2.08 (−2.27, −1.89)	*t*(101) = −21.75	<0.001	−2.15

Data are presented as mean ± standard deviation (SD). Mean differences were calculated as post-intervention minus pre-intervention values. Step-length, stance-phase, and swing-phase symmetry indices were analyzed using Wilcoxon signed-rank tests, whereas contact-time symmetry index was analyzed using a paired-samples t-test. P values were adjusted using the Holm procedure within the specified gait symmetry outcome family. Effect sizes are reported according to the analysis: matched-pairs rank-biserial correlations (*r_rb_*) for the Wilcoxon outcomes and Cohen’s *d*_z_ for the contact-time symmetry index analyzed using a paired-samples t-test.

**Figure 3:**
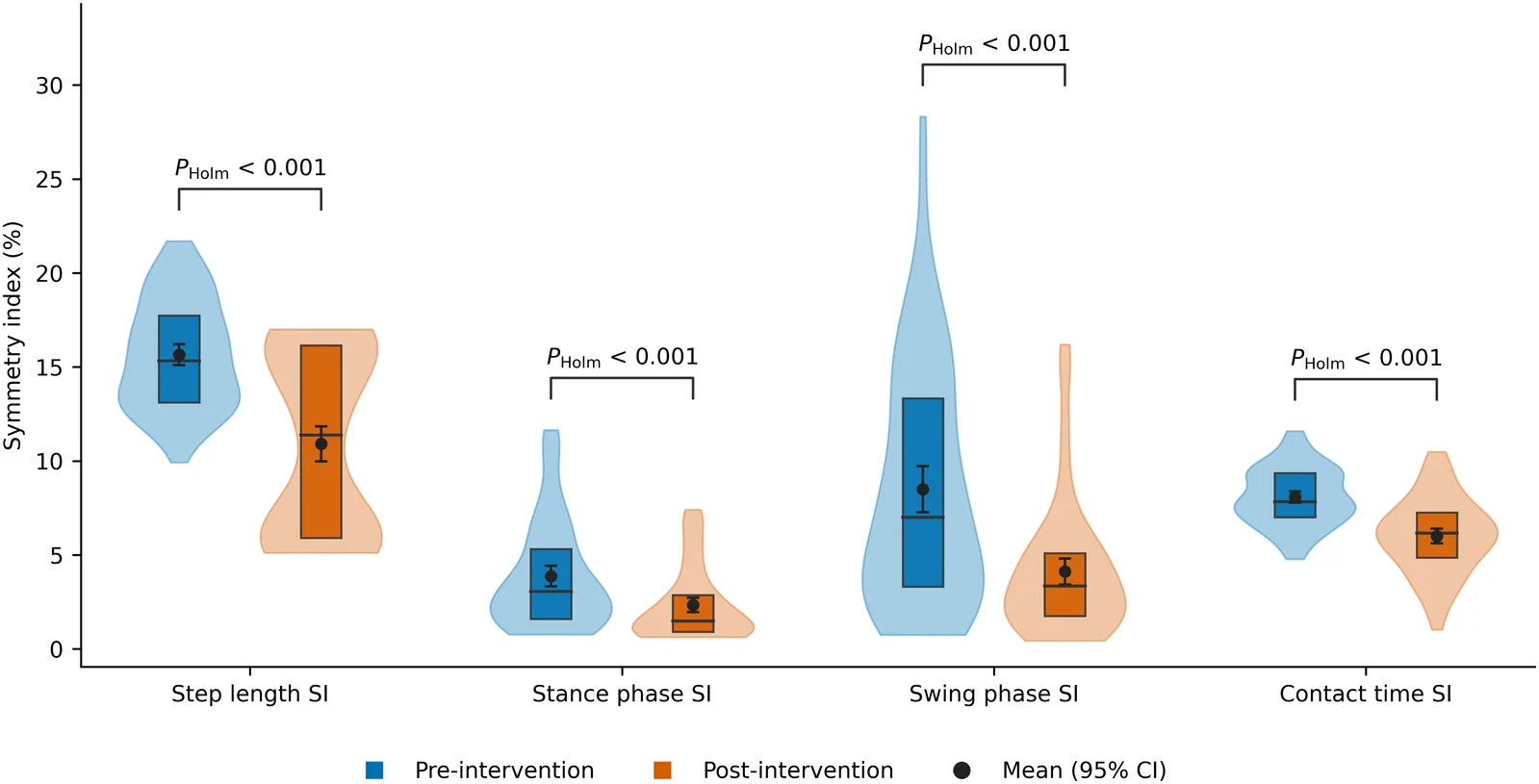
Distributions of gait symmetry indices before and after the 12-week combined exercise program among 102 completers. Step-length, stance-phase, swing-phase, and contact-time symmetry indices (SI) are expressed as percentages, with lower values indicating less inter-limb asymmetry. Blue and orange indicate pre- and post-intervention assessments, respectively. Violin shapes represent estimated distributions, boxes represent the interquartile range, horizontal lines indicate medians, and black points indicate means. Error bars represent approximate 95% confidence intervals for the mean, calculated as mean ± 1.96 × standard error; these intervals do not represent paired mean changes. Brackets indicate within-participant pre–post comparisons for each symmetry index. Step-length, stance-phase, and swing-phase symmetry indices were analyzed using Wilcoxon signed-rank tests, whereas contact-time symmetry index was analyzed using a paired-samples *t*-test. Holm adjustment was applied across the four gait symmetry outcomes; all adjusted *P* values were < 0.001.

Specifically, the step-length symmetry index decreased from 15.66 ± 2.86% to 10.91 ± 4.82% (mean difference: −4.75%, 95% CI: −5.32 to −4.13; P < 0.001). The stance-phase symmetry index decreased from 3.88 ± 2.84% to 2.35 ± 1.95% (mean difference: −1.53%, 95% CI: −1.80 to −1.27; P < 0.001). Similarly, the swing-phase symmetry index decreased from 8.50 ± 6.31% to 4.12 ± 3.57% (mean difference: −4.38%, 95% CI: −4.98 to −3.79; P < 0.001). The contact-time symmetry index also decreased significantly, from 8.09 ± 1.55% to 6.01 ± 2.03% (mean difference: −2.08%, 95% CI: −2.27 to −1.89; P < 0.001).

Overall, these findings indicate reduced gait asymmetry following the intervention. The step-length symmetry index decreased to 10.91% at the post-intervention assessment, indicating reduced step-length asymmetry.

## 4 Discussion

### 4.1 Changes in sit-to-stand performance in older adults with sarcopenia

Older adults with sarcopenia often exhibit impaired sit-to-stand performance because of reduced lower-limb muscle strength, diminished dynamic postural control, and impaired neuromuscular coordination (Sadeh et al., 2023). In the present study, the FTSST, TUGT, and STS transfer time were used to evaluate lower-limb functional performance before and after the intervention (Hyodo et al., 2023). Significant improvements were observed in all three functional outcomes following the 12-week elastic band resistance training program, suggesting improved functional mobility and task-specific motor performance in this population.

The present findings are consistent with previous studies investigating elastic band-based resistance training in older adults. Huang et al. reported that progressive elastic band resistance exercise improved body composition and physical function in older women with sarcopenic obesity (Huang et al., 2017), whereas Liao et al. demonstrated improvements in lean mass and physical capacity following elastic band training in a similar population (Liao et al., 2018). More recently, Meng et al. demonstrated that elastic band resistance training significantly improved lower-limb strength, chair-stand performance, functional mobility, and balance in older adults, further supporting the present findings (Meng et al., 2025). Collectively, these findings support elastic band resistance training as a practical and potentially effective intervention for improving physical function in older adults with reduced muscle function. Compared with conventional machine-based resistance training, elastic band training requires minimal equipment and training space while allowing progressive resistance adjustment, making it particularly suitable for community- and home-based exercise programs (Biben et al., 2019; Lopes et al., 2019). In the present study, shorter FTSST, TUGT, and STS transfer times indicate better performance on these tasks at the post-intervention assessment. Changes in lower-limb strength or neuromuscular coordination are possible explanations, but these mechanisms were not directly tested and cannot be established from this uncontrolled comparison.

Elastic band resistance training has been widely recognized as an effective strategy for improving muscle strength and physical function in older adults (Valdés-Badilla et al., 2023). Previous studies have shown that regular resistance exercise improves postural control, lower-limb coordination, joint flexibility, and walking performance in older adults with sarcopenia (Liao et al., 2018). These adaptations may facilitate more efficient interaction between the neuromuscular and musculoskeletal systems during functional tasks, thereby improving movement coordination (Tsai et al., 2022). In addition, strengthening key lower-limb muscles, particularly the quadriceps and plantar flexors, may contribute to improved sit-to-stand performance and functional mobility (Zhao et al., 2022). A meta-analysis reported beneficial effects of elastic band training on physical function in older adults with sarcopenia (Tsai et al., 2022).

These findings are consistent with the functional benefits of elastic band training reported in older adults with sarcopenia (Tsai et al., 2022); whether improved neuromuscular coordination underlies these benefits remains uncertain. A 12-week elastic-band training study in community-dwelling older adults reported improvements in handgrip strength and six-minute walking distance (Biben et al., 2019). Silva et al. likewise reported that 12 weeks of progressive resistance training reduced sit-to-stand and Timed Up and Go completion times while improving muscle strength and overall physical function in older adults with sarcopenia (Silva et al., 2023). Together, these findings suggest that resistance training is associated with improvements in lower-limb functional performance and dynamic mobility in older adults.

Overall, the observed reductions in FTSST, TUGT, and STS transfer time indicate better performance on these functional tasks at the post-intervention assessment among participants who completed both assessments. Because the program also included single-leg balance training and body-weight heel raises, these components may have contributed to the observed functional improvements; their individual effects cannot be isolated in this single-arm combined-program design.

### 4.2 Changes in task-related EMG indices (RMS and IEMG)

To evaluate the second hypothesis, neuromuscular activation was assessed using RMS and IEMG obtained from key lower-limb muscles during the STS task. Following the intervention, task-normalized RMS values decreased significantly in all examined muscles, whereas significant increases in IEMG were observed in the bilateral rectus femoris and tibialis anterior muscles. No significant changes were detected in gastrocnemius IEMG after Holm adjustment. These findings suggest training-associated changes in task-related EMG-derived activation indices during STS performance.

Thapa et al. reported significant within-group reductions in five-times sit-to-stand and Timed Up and Go completion times after four weeks of resistance exercise, with or without electrical muscle stimulation, in middle-aged and older women; however, between-group differences in change were not statistically significant (Thapa et al., 2023).

The significant decrease in task-normalized RMS observed across all examined muscles indicates lower task-normalized RMS values during STS following the intervention. In contrast, significant increases in IEMG were observed only in the bilateral rectus femoris and tibialis anterior muscles, whereas the gastrocnemius muscles did not exhibit statistically significant changes after Holm adjustment. During the STS task, the knee extensors play an important role in generating the joint moments required to elevate the body, whereas the tibialis anterior contributes to anticipatory postural adjustment, forward displacement of the center of mass, and stabilization during the transition from sitting to standing (Sadeh et al., 2023). The more pronounced changes observed in the rectus femoris and tibialis anterior may therefore reflect their task-specific involvement in the STS movement. By comparison, the absence of a significant change in gastrocnemius IEMG may reflect differences in muscle-specific functional demands during this task; however, this interpretation remains speculative because individual muscle contributions and neuromuscular strategies were not directly assessed. Accordingly, these observations should be interpreted as changes in EMG-derived activation indices rather than as direct evidence of enhanced neuromuscular function.

Several previous studies provide relevant context for the present findings. Bellomo et al. reported improvements in physical function and balance following resistance training in older adults with sarcopenia (Bellomo et al., 2013). Watanabe et al. demonstrated that a 12-week very low-intensity resistance training program improved quadriceps muscle size and knee-extension strength in healthy older adults (Watanabe et al., 2014). Similarly, Shin et al. reported that a 12-week strength-training program was accompanied by a significant increase in integrated EMG, suggesting training-associated changes in muscle activation (Shin et al., 2006). Leone et al. summarized the application of surface EMG for characterizing neuromuscular features in individuals with sarcopenia (Leone et al., 2025). In addition, a meta-analysis by Tsai et al. demonstrated that elastic band training significantly improved grip strength, gait speed, functional mobility, and appendicular skeletal muscle index in older adults with sarcopenia (Tsai et al., 2022). Collectively, these studies support the broader functional benefits of resistance training and provide context for the training-associated changes in EMG-derived activation observed in the present study.

Taken together, the observed changes in RMS and IEMG indicate that the intervention was associated with changes in task-related EMG-derived activation indices during the STS task. However, these findings should be interpreted cautiously because the present study did not directly assess muscle strength, motor-unit recruitment, or the physiological mechanisms underlying the observed EMG changes.

An additional methodological consideration should be acknowledged when interpreting the EMG findings. In the present study, task-normalized RMS was calculated using a resting-baseline-corrected task-related reference rather than an external MVIC. The resulting ratio expresses the baseline-corrected whole-epoch RMS relative to the baseline-corrected within-task peak RMS. Because the reference was obtained separately at each assessment, a lower ratio may reflect a change in the whole-epoch RMS, the peak reference, or both; it does not by itself demonstrate reduced absolute EMG amplitude or improved neuromuscular efficiency. These values should not be interpreted as direct measures of absolute muscle activation capacity. Furthermore, IEMG was analyzed in its original amplitude-related unit, and surface electrodes were re-applied at the post-intervention assessment. Consequently, between-session differences in EMG amplitude may have been influenced not only by physiological adaptations but also by variations in electrode placement, skin–electrode impedance, and other measurement-related factors. Accordingly, the EMG findings should be interpreted as training-associated changes in EMG-derived task indices rather than as direct evidence of altered muscle activation capacity. Future studies incorporating standardized MVIC normalization procedures together with more consistent electrode placement protocols across repeated assessments are warranted to improve the comparability of longitudinal EMG measurements.

### 4.3 Changes in balance function

The Berg Balance Scale (BBS) was used to evaluate balance function in older adults with sarcopenia. Following the 12-week elastic band resistance training program, BBS scores increased significantly, indicating better balance performance after the intervention. These findings are consistent with previous studies demonstrating that resistance-based exercise can improve balance and physical function in older adults and individuals with sarcopenia (Liang et al., 2020; Keating et al., 2021; Zhao et al., 2022).

BBS scores were higher after the combined exercise program. Elastic-band exercises, single-leg balance training, and body-weight heel raises may each have contributed, but their individual contributions cannot be separated in this design. Changes in motor control or force-generating capacity remain possible mechanisms rather than demonstrated findings. Previous studies have similarly reported that elastic band resistance training improves physical function, balance, gait speed, and lower-limb functional performance in older adults with sarcopenia or sarcopenic obesity (Liao et al., 2018; Valdés-Badilla et al., 2023; Meng et al., 2025).

Higher BBS scores indicate better performance on the balance assessment at the post-intervention assessment. This study did not directly evaluate sensorimotor integration or establish whether these changes translated into fewer falls or greater functional independence. These potential clinical implications require further investigation.

### 4.4 Changes in gait symmetry and bilateral coordination

Gait symmetry reflects the degree of bilateral consistency during walking and is widely regarded as an important indicator of gait quality and locomotor function (Pizzigalli et al., 2011; Patterson et al., 2012). Previous studies have shown that gait asymmetry tends to increase with advancing age and has been associated with impaired mobility, reduced balance performance, and an increased risk of falls (Laroche et al., 2012; Guadagnin et al., 2019; Shafizadeh et al., 2023). Therefore, improving gait symmetry represents an important rehabilitation goal for older adults with sarcopenia.

In the present study, gait symmetry was evaluated using four commonly reported temporal and spatial parameters, including the step-length symmetry index, stance-phase symmetry index, swing-phase symmetry index, and contact-time symmetry index. Following the 12-week elastic band resistance training program, all four gait symmetry indices were significantly reduced, indicating reduced inter-limb gait asymmetry during walking.

The observed changes may be related to improvements in lower-limb functional capacity, dynamic balance, and postural control following repeated resistance and functional exercise. Previous research has shown that lower-limb muscular strength and bilateral symmetry are relevant to postural stability in older adults, whereas resistance training may improve gait- and balance-related outcomes in this population (Pizzigalli et al., 2011; Keating et al., 2021). A systematic review reported associations between functional performance and gait asymmetry in older adults, while the relationship between muscular performance and gait asymmetry remained uncertain (Guadagnin et al., 2019). Improved lower-limb function and dynamic balance may therefore facilitate more symmetric weight transfer and temporal regulation between limbs during walking. However, because lower-limb kinetics, muscle coordination strategies, and central motor control were not directly assessed in the present study, these potential mechanisms remain speculative. The single-leg balance and body-weight heel-raise components may also have contributed to the observed gait changes, but the present design cannot distinguish their effects from those of the elastic-band exercises.

The lower symmetry indices observed after the program indicate reduced inter-limb asymmetry in the measured gait parameters. However, these changes do not establish the underlying neuromuscular mechanisms, and the uncontrolled study design limits attribution of the observed changes to the training program. The remaining asymmetry may reflect persistent age- and sarcopenia-related limitations in lower-limb strength, motor control, or gait adaptability (Guadagnin et al., 2019; Shafizadeh et al., 2023). Whether a longer intervention period or greater training intensity could further improve gait symmetry warrants investigation in future studies.

Overall, the present findings suggest that elastic band resistance training was associated with improvements in gait symmetry indices and reduced gait asymmetry during walking in older adults with sarcopenia. However, because this was a single-arm intervention study, randomized controlled trials incorporating direct biomechanical and neuromuscular measurements are needed to clarify the mechanisms and persistence of the observed changes.

## 5 Limitations

This study has several limitations. First, the single-arm pre–post design without a concurrent control group limits causal inference and cannot rule out placebo effects, Hawthorne effects, regression to the mean, or changes partly related to repeated testing. The program was delivered through a college–community service partnership and offered to all eligible community participants. Under this service-delivery arrangement, a concurrent group not receiving the program was not established. This explains the practical context of the single-arm design but does not overcome its limitations for causal inference. In addition, the program combined elastic-band exercises with single-leg balance training and body-weight heel raises; the contributions of these components cannot be separated. Future community-based studies could use a wait-list control, stepped-wedge design, or cluster-randomized design so that all participants eventually receive the intervention while allowing stronger causal inference. A further limitation is that a prospective hierarchy of primary and secondary outcomes was not explicitly documented. The use of FTSST in the reported pilot-based sample-size calculation does not, by itself, establish its prospective designation as the primary outcome. The analyses should therefore be interpreted as exploratory, and within-family multiplicity adjustment does not eliminate the possibility of selective emphasis across outcomes.

Second, although 120 participants were initially recruited, 102 completed the 12-week intervention and post-intervention assessments, resulting in an attrition rate of 15.0%. The 18 non-completers attended fewer than 29 sessions; their reasons for non-completion were personal reasons (n = 6), illness causing mobility difficulties (n = 5), and loss to follow-up (n = 7). Because the pre–post outcome analyses were based on complete cases rather than an intention-to-treat approach, differential adherence and attrition may have introduced potential bias and may reduce the robustness of the findings. Future studies should predefine strategies for handling missing data and conduct intention-to-treat or sensitivity analyses to examine the influence of attrition on intervention effects.

Third, adherence was assessed using attendance logs maintained by on-site trainers and weekly self-reported training diaries, but attendance does not quantify the exercise intensity achieved by individual participants. No formal rating-of-perceived-exertion scale was recorded, limiting the characterization of the delivered training intensity. The relationship between the illness-related mobility difficulties and the exercise intervention could not be determined from the available analysis dataset.

Fourth, sex- and age-related differences in training responses were not examined. Although the final sample included both men and women, the study was not specifically powered or designed to compare whether improvements in sit-to-stand performance, lower-limb skeletal muscle activation, balance function, or gait symmetry differed by sex or age subgroup. Therefore, potential sex- and age-specific adaptations to the 12-week resistance band training program remain unclear. Future studies with larger samples should conduct stratified or interaction analyses to clarify whether sex and age modify the effects of elastic band training in older adults with sarcopenia.

Finally, this was a single-center study involving a relatively homogeneous sample of community-dwelling older adults with sarcopenia, which may limit the generalizability of the findings to broader older-adult populations, clinical populations, or individuals with different levels of frailty. Long-term follow-up was also not conducted; therefore, the sustainability of the observed training effects remains unclear. Future studies should include multicenter samples, objective or technology-assisted adherence monitoring, and follow-up assessments to determine whether the benefits of elastic band training can be maintained over time.

## 6 Conclusion

Among the 102 participants who completed both baseline and post-intervention assessments, sit-to-stand performance and balance scores improved between baseline and the end of the 12-week combined exercise program. Lower gait symmetry indices were observed following the program, consistent with reduced inter-limb asymmetry during walking. Task-normalized RMS values were lower across the examined muscles, whereas IEMG changes were muscle-specific. The absence of a concurrent control group prevents attribution of the observed changes to the program or its individual components. Attrition and limitations of between-session EMG comparisons, including the absence of independent MVIC normalization, further constrain interpretation. Controlled studies are needed to investigate training effects and underlying mechanisms and to assess the persistence of the observed changes.

## Data Availability

The raw data supporting the conclusions of this article will be made available by the authors, without undue reservation.
