# Supplementary material for: Changes in functional performance, lower-limb muscle activation, balance, and gait symmetry after a 12-week elastic band resistance training program in older adults with sarcopenia: a single-arm pre–post study
Source: Front Physiol. 2026 Oct 29;17:1970041. doi: 10.3389/fphys.2026.1970041 (PMC13754746; doi:10.3389/fphys.2026.1970041)
Supplement: Supplementary file 1 [file Table1.docx]

**Supplementary Table S1. Elastic band-based resistance training protocol used in the 12-week intervention**

| **No.** | **Exercise** | **Main target** | **Equipment and resistance adjustment** | **Training dose** | **Progression and safety criteria** | **Functional relevance** |
| --- | --- | --- | --- | --- | --- | --- |
| 1 | **Sideways Bow Drawing** | Shoulder girdle, upper back, trunk rotators, postural control | Elastic band. Initial resistance generally started with the yellow band during familiarization; resistance was adjusted using band color, band length, elongation, and grip position. | 2 sets × 10–15 repetitions per side | Progression was allowed when participants completed the prescribed repetitions with correct technique and without excessive fatigue, pain, or compensatory movement. Resistance was reduced if poor movement control, discomfort, or excessive fatigue occurred. | Enhances trunk control and upper-limb coordination, supporting postural stability during transitional movements. |
| 2 | **Chest Push-Out** | Pectoral muscles, anterior shoulder, triceps, trunk stabilization | Elastic band placed behind the back. Resistance was adjusted using band color, band length, elongation, and grip position. | 2 sets × 10–15 repetitions | Same progression and safety criteria as above. | Improves upper-body support capacity and trunk stability, which may assist balance control during functional mobility tasks. |
| 3 | **Lateral Shoulder Abduction** | Deltoid, shoulder stabilizers, upright postural control | Elastic band fixed under the ipsilateral foot. Resistance was adjusted using band color, band length, elongation, and grip position. | 2 sets × 10–15 repetitions per side | Same progression and safety criteria as above. | Promotes postural control during standing while challenging upper-limb movement coordination. |
| 4 | **Standing Hip and Knee Flexion with Band Resistance** | Hip flexors, knee flexors, tibialis anterior, dynamic balance | Elastic band secured around both ankles or fixed by the opposite foot/ankle. Resistance was adjusted using band color, band length, elongation, and movement range. | 2 sets × 10–15 repetitions per side | Same progression and safety criteria as above. Light external support was allowed when needed for safety. | Trains limb advancement, foot clearance, and dynamic balance, which are relevant to gait initiation and walking symmetry. |
| 5 | **Seated Knee Extension** | Quadriceps, especially rectus femoris | Elastic band secured around both ankles. Resistance was adjusted using band color, band length, elongation, and knee range of motion. | 2 sets × 10–15 repetitions per side | Same progression and safety criteria as above. | Strengthens the quadriceps, a key muscle group for sit-to-stand performance and knee stability during stance. |
| 6 | **Standing Hip Extension** | Gluteal muscles, hamstrings, posterior chain, postural control | Elastic band secured around both ankles. Resistance was adjusted using band color, band length, elongation, and hip range of motion. | 2 sets × 10–15 repetitions per side | Same progression and safety criteria as above. Light external support was allowed when needed for safety. | Enhances hip extensor strength and posterior-chain control, supporting standing-up ability, propulsion, and gait stability. |
| 7 | **Single-Leg Stand** | Static balance, hip abductors, ankle stabilizers, proprioception | Body weight. No elastic band was used. Stable support was allowed for safety if needed. | 2 sets × 2–2.5 min per leg | Participants were encouraged to reduce hand support as balance improved, while maintaining safety and correct posture. | Improves single-limb support capacity and postural stability, which are essential for stance-phase control and gait symmetry. |
| 8 | **Standing Heel Raises** | Gastrocnemius, soleus, ankle plantarflexors, ankle stability | Body weight. No elastic band was used. Stable support was allowed for safety if needed. | 2 sets × 20 repetitions | Progression emphasized controlled movement, full available range, and reduced external support when safe. | Strengthens the ankle plantarflexors and improves push-off control, contributing to gait propulsion and temporal symmetry. |

**Note.** Note. Yellow, red, green, and blue bands provided progressively greater resistance, with approximate manufacturer-reported ranges of 0.5–1.3, 1.3–2.6, 2.6–4.5, and 4.5–7.2 lbs, respectively, at 100% elongation. Available lengths were 8 inches (20 cm), 12 inches (30 cm), and 18 inches (46 cm). Yellow bands were used initially during familiarization; subsequent resistance was individualized.

Groups of approximately 10–12 participants were supervised by one instructor and one assistant. Training intensity was adjusted according to participant feedback, movement quality, and repetition completion. Repetitions were increased within the 10–15 range before resistance was increased through adjustments to band elongation, grip length, band length, or color. Progression required good movement control and acceptable tolerance; resistance or repetitions were reduced when pain, excessive fatigue, or compensatory movements occurred.

Exercises 7 and 8 used body weight only, with doses and progression criteria specified in the table. No formal Borg RPE scale, 0–10 RPE scale, or visual analog scale was recorded.
